# Evaluating Simulation-Based Tobacco Treatment Scenarios for Providers Delivering Treatment for People Living With Mental Illnesses

**DOI:** 10.3389/fpsyt.2022.868550

**Published:** 2022-04-06

**Authors:** Chizimuzo T. C. Okoli, Janet K. Otachi, Sarret Seng, Bassema Abufarsakh, Lovoria B. Williams

**Affiliations:** ^1^College of Nursing, University of Kentucky, Lexington, KY, United States; ^2^College of Social Work, University of Kentucky, Lexington, KY, United States

**Keywords:** tobacco treatment, animated scenarios, mental health, behavioral health (BH) patients, substance use

## Abstract

**Background:**

People living with mental illnesses (PMI) experience elevated tobacco use and related morbidity and mortality. Despite the availability of effective and safe tobacco treatments along with evidence that PMI are motivated and able to quit successfully, few Mental and behavioral healthcare providers (MHPs) engage PMI in such treatment. MHPs may lack the confidence or skills to engage their clients in tobacco treatment. Currently, there are limited training modalities to prepare MHPs in delivering tobacco treatment for PMI. However, animated scenario-based simulated encounters can bridge this gap to effectively provide tailored MHP training to enhance treatment delivery. Hence, the purpose of this study was to evaluate simulated tobacco treatment education scenarios tailored to MHPs.

**Methods:**

For this evaluation, we used a pretest-posttest design to assess changes in MHPs tobacco treatment knowledge and behavioral intentions after viewing simulated treatment encounters. We developed four animated scenarios, using brief tobacco treatment interventions, simulating treatment encounters with PMI. MHPs were primarily recruited from mental or behavioral healthcare facilities and were asked to complete a web-based questionnaire. Their knowledge, views, and experiences in providing tobacco treatment were assessed prior to viewing the animated scenarios. Participants were then asked to evaluate the desirability, acceptability, and applicability of the animated scenarios; and thereafter, their knowledge of and intentions to provide evidence-based tobacco treatment (i.e., ASK, ADVISE, ASSESS, ASSIST, ARRANGE) were again assessed.

**Results:**

Participants (*N* = 81) were on average 41.0 years of age, mostly female (79.0%), and non-Hispanic White (86.4%). Nearly a quarter endorsed current tobacco use and few had tobacco treatment training (14.8%). Overall knowledge of tobacco treatment scores significantly increased before and after viewing the videos (*M* = 3.5 [SD = 1.0] to *M* = 4.1 [SD = 1.0], *p* < 0.0001). After viewing the simulated scenario videos, participants endorsed moderate to high mean scores (ranging from 4.0-4.2 on a 0 to 5 scale) on the desirability, acceptability, and applicability of the different animated scenarios. In addition, after viewing the scenarios the proportion of participants who endorsed that they intended to occasionally/very often engage clients in evidence based tobacco treatment were high for ASK (94.9%), followed by ADVISE and ASSESS (84.7% each), followed by ASSIST (81.4%), and ARRANGE (74.6%). Evaluation scores significantly differed by type of animated scenario and participants' work settings and discipline.

**Conclusions:**

These findings suggest that the use of brief animated scenarios may be a useful modality to enhance MHPs knowledge acquisition and treatment delivery intentions. Such approaches may be integrated into tobacco treatment trainings for MHPs.

## Introduction

After over 50 years of promoting and testing tobacco control efforts in the United States (U.S.), there is equivocal science on what is most essential for successful tobacco control. These essential elements, summed up in the pillars of tobacco control endorsed by the Centers for Disease Control and Prevention (CDC), include preventing initiation, promoting cessation, eliminating secondhand tobacco smoke exposure, and de-normalizing tobacco use as a behavior (1). In terms of promoting cessation, healthcare delivery systems are strongly encouraged to adopt evidence-based tobacco treatment practices. These practices include multi-faceted approaches that support consumers by providing tobacco cessation pharmacotherapy, supporting behavioral counseling, and enacting organizational policies that promote best practices, such as tobacco-free policies (2). Adopting such strategies has been instrumental in curbing tobacco prevalence in the U.S., reducing the percentage of adult smokers from about 25% in 2002 to 14% in 2019 (3, 4).

Unfortunately, adoption of proven evidence-based practices has been particularly challenging within the mental and behavioral healthcare system in the U.S. Due to these gaps in integrating evidence-based tobacco treatment approaches within mental healthcare systems, people living with mental illnesses (PMI) experience disproportionate tobacco use prevalence, morbidity, and mortality as compared to the general population (5). For example, compared to people without mental illnesses, PMI have 2-3 times the tobacco use prevalence, higher rates of cardiovascular and lung disease, and die on average 10–25 years prematurely (5–8).

These disproportionate tobacco-related challenges among PMI persist despite the increasing evidence of the benefits associated with tobacco cessation on mental health outcomes (9). In fact, only 48.6% of mental healthcare systems in the U.S. have smoke-free policies and only 21.5–48.9% have treatment policies supporting evidence-based tobacco cessation interventions (10–12). Moreover, the delivery of evidence-based tobacco treatment within mental healthcare settings faces multi-faceted challenges including patient barriers (e.g., stressors that are relieved by tobacco use), mental healthcare provider (MHP) barriers (e.g., being poorly equipped to provide tobacco treatment and believing patients are not interested in quitting) and organizational barriers (e.g., lack of training for clinicians and staff) (13–15). Therefore, examining approaches that facilitate provider delivery of tobacco treatment may guide the development of effective strategies to enhance tobacco treatment engagement for PMI.

Prior research suggests that provider delivery of evidence-based tobacco treatment can be enhanced through targeted training (14, 16). In fact, targeted training in tobacco treatment increases healthcare providers' confidence in and delivery of tobacco treatment (17). Animated scenario-based simulated encounters may be an effective method to provide tobacco treatment education in mental health settings (18, 19). Simulation-based trainings may have the advantage of reaching a wide audience through cost-effective and resource efficient means, as compared to traditional face-to-face trainings (20–22). Developing and evaluating such simulated encounters can demonstrate their utility as training tools for MHPs.

The purpose of this study was to evaluate simulated tobacco treatment education scenarios tailored to MHPs. Specifically, we aimed to:

1) Assess providers' ratings on the desirability, applicability, and acceptability of simulated tobacco treatment scenarios, and
2) Examine changes in provider knowledge of tobacco use and treatment among PMI after engaging in the simulated treatment scenarios, and
3) Determine provider intentions to provide evidence-based tobacco treatment after engaging in the simulated treatment scenarios.

This evaluation may guide future research and practice regarding the use of simulated scenarios as tools for MHP tobacco treatment training.

## Methods

### Study Design

This evaluation study employed a single-group pre/post-test design to examine changes in provider knowledge about tobacco use treatment among PMI after engaging in simulated scenario videos. In addition, a post-test only design was used to examine providers' intentions regarding delivery of evidence-based tobacco treatment after watching the videos. A targeted sample of MHPs for the study was obtained through purposive sampling.

### Study Population

Our research team contacted Key leadership within the Community Mental Health Centers (CMHCs) and targeted behavioral healthcare organizations to request permission to recruit providers for the evaluation. To determine the utility of the scenarios across disciplines and roles we targeted four different disciplines: prescribers, (e.g., physicians and nurse practitioners), counselors/therapists, nurses, and social workers for information. Our recruitment goal was 80 providers with a minimum of five from each discipline and role to obtain an estimated 20 providers per scenario. We recruited providers from 13 CMHCs, two outpatient behavioral health treatment programs, two inpatient behavioral health programs, and one substance use treatment programs for women. To support survey completion, the main contacts from each organization were sent an email reminder every two weeks throughout the data collection timeframe from June 1st to October 31st, 2021.

### Intervention

Certified tobacco treatment specialists with extensive experience treating PMI and training other healthcare providers developed the four scenarios. Each scenario was developed to simulate the experience of an initial tobacco treatment encounter with a PMI. The scenarios were further tailored to specific PMI populations based on our extensive work on exploring the unique cessation needs voiced by PMI and MHPs who deliver care to them (14, 23–27). We then obtained face validity of the scenarios through review by other tobacco treatment specialists and healthcare providers for PMI.

Each scenario was ~22–27 min in duration and comprised of two parts. Part A consisted of a 2–3 min general information regarding the prevalence of factors associated with tobacco use in specific PMI and treatment approaches for addressing tobacco dependence. The specific PMI populations were: Attention Deficit Hyperactivity Disorder (ADHD), Schizophrenia Spectrum Disorder (SSD), Major Depressive Disorder (MDD), and Substance Use Disorders (SUDs). Part B consisted of a 20–24 min animated scenario of a provider engaging a PMI in evidence-based tobacco treatment modeled after the 5As (Ask, Advise, Assess, Assist, Arrange) framework (28). Special care was taken to include diversity in terms of gender, age, ethnicity, and type of setting (i.e., inpatient psychiatric setting vs. outpatient setting) when developing the scenarios. The four scenarios can be viewed here:

1) SSD: https://www.youtube.com/watch?v=Tor9OIg5Ap0&list=PLYHtV_ZWwXeBtPD5ZjFRNLcjnQl24KR5E&index=4&t=0s
2) MDD: https://www.youtube.com/watch?v=_loAkQ3zDzM&list=PLYHtV_ZWwXeBtPD5ZjFRNLcjnQl24KR5E&index=6
3) ADHD: https://www.youtube.com/watch?v=g1BgpvVBVGI&list=PLYHtV_ZWwXeBtPD5ZjFRNLcjnQl24KR5E&index=4
4) SUDs: https://www.youtube.com/watch?v=YGxxyW1Bzxw&list=PLYHtV_ZWwXeBtPD5ZjFRNLcjnQl24KR5E&index=3~

### Procedure

Approval was obtained from the relevant institutional boards governing the conduct of research with human subjects. We then sent a standardized email containing a link to the administrative staff of the participating organizations, who then sent the link through provider listservs. Participants were re-assured that their responses, recorded through the encrypted survey platform Qualtrics were anonymous. For those who indicated interest in participating, an informed consent form with detailed information about the study was provided to participants. If the participant clicked on “I consent” after reading the consent form, they were directed to the survey. Completion of the evaluation survey was ~30–40 min, comprising of a pre-test questionnaire (~5–7 min), the scenario (~22–27 min), then completing a post-test questionnaire (~5 min). Participants who completed the survey and evaluated the simulated scenarios were provided a $50 incentive.

To obtain a diversity of opinions while considering the salience of each scenario, we randomly distributed the four scenarios among the CMHCs to obtain information from this unique context. Each scenario was further sent to targeted settings given the specific population of interest in the scenario. For example, the scenario which depicted tobacco treatment with a patient with SSD being discharged from an inpatient hospital stay was sent to MHPs at two inpatient psychiatric settings and the scenario in which an adolescent with ADHD is being treated for use of juuls was sent to MHPs in a behavioral health program that specializes in the treatment of children, adolescents, and families. Furthermore, the scenario which depicts a young lady with MDD was sent to MHPs in outpatient behavioral health programs. Finally, the scenario of a young mother who has SUD was also sent to MHPs serving at residential substance use treatment programs for mothers.

### Measures

Demographic data included age, sex, education (less than college vs. college graduate), race and ethnicity.

#### Provider and Practice Characteristics

Provider and practice characteristics included information on job role (Prescriber [MD/APRN], Nurse [LPN/RN] Social Worker [LSW/LCSW] vs. Psychologist/Counselor [LPCC/LMFT] vs. other), practice setting (CMHC vs. Outpatient Behavioral Health/Residential Recovery vs. Inpatient Behavioral Health vs. other), primary population served (adults vs. pediatrics vs. women) and work tenure in months.

#### Tobacco Use and Treatment Experience

Participants were also asked about their tobacco use in the last 30 days (yes vs. no), receipt of prior tobacco treatment training (yes vs. no), and the frequency of their delivery of the 5As on a scale of 0 = never to 3 = very often. For analysis, the frequency of delivery of each component of the 5As were categorized into 0 = never/seldom and 1 = occasionally/very often.

#### Desirability, Applicability, and Acceptability Ratings of Animated Simulated Tobacco Treatment

To assess each component of the simulated scenario, we used measures similar to those used in a previous study (23). We asked the following question:

“We would like your opinions about this video we have developed to train providers on evidence-based tobacco treatment tailored to a patient/client living with [specific mental illness]. We would like you to rank the video on a scale of 0 to 5, based on how much you see it as desirable, applicable, and acceptable to you and other providers caring for people with [specific mental illness]. We explained desirable as “something you would want to hear/learn about”; applicable as “something that is useful to you/you could use” and acceptable as “something that would gain your interest/would make you seek more information”. The mean scores on the desirability, applicability, and acceptability ratings of the information component (part A), the evidence-based tobacco treatment components (part B), and an assessment of the use of animation were used to evaluate the simulated scenarios.

#### Knowledge of Tobacco Use and Treatment in Specific PMI Populations

For each scenario, a five-item knowledge questionnaire was developed with ‘true/false' response choices to elicit specific information provided in the part A of each scenario. For example, for the SSD scenario, the questions were as follows:

1. People with schizophrenia are equally as likely to use tobacco as compared to the general population.
2. Nicotine from tobacco use is not addictive and causes agitation for people with schizophrenia.
3. People with schizophrenia are more likely to use tobacco because of permissive attitudes of providers to tobacco use within behavioral health settings.
4. People with schizophrenia are unable to stop using tobacco because no evidence exists in ways to help them.
5. Tobacco use can reduce the effectiveness of medications used to treat schizophrenia.

Each question was tailored to the specific MI addressed in the corresponding video. The same questions were asked before and immediately after participants watched the videos. Mean summary scores of the questions were obtained. Also, proficiency measurements were derived by determining individuals scoring 4 of 5 questions correct (i.e., 80%).

#### Intentions to Provide Evidence-Based Tobacco Treatment

After watching the videos, participants were asked about their intentions to provide tobacco treatment using the 5 As model as follows: “In your practice role, how often do you anticipate that you will:

1. ASK patients/clients whether they smoke cigarettes or use other tobacco products?
2. ADVISE patients/clients who smoke or use tobacco products to quit?
3. ASSESS the readiness of patients/clients who smoke or use other tobacco products to quit or cut down?
4. ASSIST patients/clients in stopping smoking/tobacco use by providing medications and/or counseling?
5. ARRANGE for patients/clients to be referred to smoking/tobacco use cessation services or follow up with them on their abstinence?

Each question had a response choice of 0 = never to 3 = very often. For analysis, the frequency of anticipated delivery of each component of the 5As were categorized into 0 = never/seldom and 1 = occasionally/very often.

### Data Analysis

Eighty-one participants provided response to the main outcomes of the evaluation survey. Of these respondents, 77 (95.1%) provided an evaluation of the scenarios and 59 (72.8%) provided complete responses to both the pre-test and post-test questions. Moreover, three participants did not provide their age, but provided their years of practicing in the discipline. Conservatively, we estimated their age by assuming they started practicing in their discipline at the age of 22. For example, a respondent who indicated that they had practiced in the discipline for 27 years but did not provide their age was assumed to be 49 years of age.

Descriptive statistics, including means with standard deviations or frequencies with percentages, were used to describe the sample as appropriate. Differences in demographic variables by the four scenarios among respondents were examined using Chi-Square analyses or Analysis of Variance (ANOVAs). Furthermore, differences in providers' scores on knowledge and practices regarding tobacco treatment by job role and work setting were examined using chi-square analyses and ANOVAs. Providers' ratings on the desirability, applicability, and acceptability of simulated tobacco treatment scenarios were examined by scenario, job role and practice setting using ANOVAs. Changes in provider tobacco treatment knowledge and proficiency scores before and after the simulated scenario training were assessed using paired-sample *t*-tests and McNemar tests, respectively. Finally, frequencies and percentages were used to describe providers' frequency of and intentions to deliver tobacco treatment prior to and after watching the simulated scenarios. Analyses were performed using IBM-SPSS Statistics version 28 (29) with a selected significance level of alpha = 0.05.

## Results

### Sample Characteristics

Table 1 provides a description of the 81 respondents. Survey respondents were on average 40.1 (SD = 9.0) years of age and primarily female (79.0%), college graduates (96.3%), and identified as White non-Hispanic (86.4%). On average participants had worked for 101 (SD = 84.7) months in their discipline, worked in CMHCs (43.2%), were nurses (29.6%) or social workers (27.2%), and served adult populations (69.1%). Few participants had tobacco treatment training (14.8%), and nearly a quarter were current tobacco users. There were significant differences in practice setting and populations served by simulated scenarios. A larger proportion of respondents evaluating the ADHD scenario were from CMHCs and the majority of those evaluating the schizophrenia scenario served the adult population.

**Table 1:** Sample characteristics by simulated scenario.

	**Total** **(*n* = 81)**		**ADHD** **(*n* = 18)**		**Depression** **(*n* = 17)**		**Schizophrenia** **(*n* = 22)**		**SUD** **(*n* = 24)**	
	**n**	**%**	**n**	**%**	**n**	**%**	**n**	**%**	**n**	**%**
**Female**	64	79.0	11	61.1	16	94.1	16	72.7	21	87.5
**College graduate**	78	96.3	17	94.4	16	94.1	22	100.0	23	95.8
**Ethnicity/race**
White Non-hispanic	70	86.4	17	94.4	14	82.4	17	77.3	22	91.7
Black Non-hispanic	7	8.6	1	5.6	2	11.8	3	13.6	1	4.2
Asian/pacific islander	4	4.9	0	0.0	1	5.9	2	9.1	1	4.2
**Job role/License**
Prescriber^a^ (MD/APRN)	17	21.0	5	27.8	7	41.2	1	4.5	4	16.7
Nurse (LPN/RN)	24	29.6	3	16.7	3	17.6	12	54.5	6	25.0
Social worker (LSW/LCSW)	22	27.2	5	27.8	4	23.5	5	22.7	8	33.3
Psychologist/counselor (LPCC/LMFT)	12	14.8	4	22.2	2	11.8	5	18.2	2	8.3
Other (administration/peer specialist)	6	7.4	1	5.6	1	5.9	0	0.0	4	16.7
**Tobacco treatment training**	12	14.8	3	16.7	3	17.6	1	4.5	5	20.8
Practice setting^***^
CMHC	35	43.2	14	77.8	7	41.2	3	13.6	11	45.8
Outpatient behavioral health/residential recovery	11	13.6	2	11.1	3	17.6	1	4.5	5	20.8
Inpatient behavioral health	28	34.6	2	11.1	5	29.4	18	81.8	3	12.5
Other (Health Clinic/Private Practice)	7	8.6	0	0.0	2	11.8	0	0.0	5	20.8
Primarily populations^***^
Adults	56	69.1	10	55.6	13	76.5	21	95.5	12	50.0
Pediatrics	11	13.6	8	44.4	2	11.8	1	4.5	0	0.0
Women	14	17.3	0	0.0	2	11.8	0	0.0	12	50.0
**Current use of tobacco products**	18	22.2	4	22.2	4	23.5	4	18.2	6	25.0
**Age in years (M/SD)**	40.1	9.0	37.1	8.8	42.4	8.3	38.9	9.8	41.7	8.6
**Work tenure in months (M/SD)**	101.1	84.7	102.4	90.8	96.4	69.2	76.5	69.3	126.1	99.4

a *Only two physicians/psychiatrists responded to the survey among prescribers. The remaining respondents were APRNs.*

*** *p < 0.0001 (based on Chi-square analyses for categorical variables or ANOVAs for continuous variables).*

*MD, Doctor of Medicine; APRN, Advanced Practice Registered Nurse; LPN, Licensed Practical Nurse; RN, Registered Nurse; LSW, Licensed Social Worker; LCSW, Licensed Clinical Social Worker; LPCC, Licensed Professional Clinical Counselor; LMFT, Licensed Marriage and Family Therapist*.

### Provider's Ratings on the Desirability, Applicability, and Acceptability of Scenarios

Our analyses of the tobacco use and mental illness specific information per scenario revealed moderate to high scores on each of providers' desirability, applicability, and acceptability for the information about tobacco use and mental illness (i.e., part A), the evidence-based components of each of the scenarios, and the use of animation for the videos. However, mean scores were lowest on the *overall use of animation* for the Schizophrenia videos. There were significant differences in the total mean rating scores of the simulated scenarios in the ASK and ARRANGE components of the videos (see Table 2).

**Table 2:** Mean rating scores on simulated scenarios by scenario type (*n* = 77).

		**Part A:** **Information about tobacco use and mental illness**	**Part B: Evidence-based tobacco treatment components**					**Overall use of animation for the scenarios**
**Scenario**	**n**	**mean (SD)**	**ASK** **mean (SD)**	**Advise** mean (SD)^*^	**Assess** **mean (SD)**	**Assist** **mean (SD)**	**Arrange** mean (SD)^*^	**Mean (SD)**
ADHD	17	4.1 (0.8)	4.2 (0.8)	4.2 (0.8)	4.3 (0.8)	4.4 (0.7)	4.2 (0.7)	4.2 (0.9)
Depression	16	4.0 (1.0)	4.3 (0.8)	4.3 (0.7)	4.4 (0.6)	4.3 (0.6)	4.4 (0.7)	4.1 (0.8)
SSD	21	4.0 (0.6)	3.8 (0.6)	3.6 (0.7)	4.0 (0.7)	3.9 (0.6)	3.7 (0.8)	3.6 (1.0)
SUD	23	4.3 (0.5)	4.2 (0.8)	4.2 (0.8)	4.1 (0.9)	4.1 (0.8)	4.1 (0.8)	4.2 (1.1)
Total	77	4.1 (0.7)	4.1 (0.8)	4.1 (0.8)	4.2 (0.7)	4.1 (0.7)	4.1 (0.8)	4.0 (1.0)

*ADHD, Attention Deficit Hyperactivity Disorder; SSD, Schizophrenia Spectrum Disorder; SUD, Substance Use Disorder.*

* *p < 0.05*.

### Changes in Provider Knowledge of Tobacco Use and Treatment

Among participants who responded to both the pre- and post-test knowledge questions (*n* = 59), we found significant increases in knowledge and proficiency scores (see Table 3). By scenario type, there were significant improvements in knowledge scores in the schizophrenia and SUD scenarios, and proficiency scores improved in the schizophrenia scenario. By work setting, there were significant increases in knowledge and proficiency scores in the inpatient setting only. Finally, among providers, nurses had an overall significant increase in knowledge scores.

**Table 3:** Pretest and posttest knowledge and frequency of providing tobacco treatment scores by scenario, setting, and provider type (*n* = 59).

	**n**	**Knowledge score**		**Proficiency**	
**Scenario type**		**Pretest** ***M* (SD)**	**Posttest** ***M* (SD)**	**Pretest** ***n* (%)**	**Posttest** ***n* (%)**
ADHD	14	3.8 (1.1)	3.9 (1.1)	10 (71.4)	9 (64.3)
Depression	16	3.8 (0.7)	4.3 (0.8)	11 (68.8)	13 (81.3)
SSD^**^^†^	13	3.2 (1.0)	4.4 (1.1)	5 (38.5)	11 (84.6)
SUD^*^	16	3.4 (1.2)	4.0 (1.1)	8 (50.0)	11 (68.8)
**Work setting**
CMHC	25	3.9 (0.9)	4.1 (1.1)	19 (76.0)	17 (68.0)
Outpatient	10	3.5 (0.7)	4.0 (1.1)	4 (40.0)	7 (70.0)
Inpatient^***^^††^	19	3.1 (1.0)	4.2 (1.0)	8 (42.1)	16 (84.2)
Other	5	3.4 (1.5)	4.2 (0.8)	3 (60.0)	4 (80.0)
**Provider type**
Prescriber	11	3.7 (0.6)	4.2 (0.9)	7 (63.6)	8 (72.7)
Nurse^**^	18	3.3 (1.0)	4.2 (1.0)	9 (50.0)	14 (77.8)
Social worker	16	3.9 (1.0)	4.3 (1.0)	11 (68.8)	14 (87.5)
Psychologist/counselor	8	3.6 (1.2)	3.9 (1.2)	6 (75.0)	4 (50.0)
Other	6	2.8 (1.0)	3.8 (1.2)	1 (16.7)	4 (66.7)
Total^***^^†^	**59**	**3.5 (1.0)**	**4.1 (1.0)**	**34 (57.6)**	**44 (74.6)**

*Based on paired sample t-tests for knowledge scores*

* *p < 0.05;*

** *p <0.01,*

*** *p < 0.001.*

*Based on McNemar tests for proficiency scores*

† *p < 0.05,*

†† *p <0.01.*

*ADHD, Attention Deficit Hyperactivity Disorder; SSD, Schizophrenia Spectrum Disorder; SUD, Substance Use Disorder*.

### Providers Intentions to Practice Evidence-Based Tobacco Treatment

**Figure 1:**
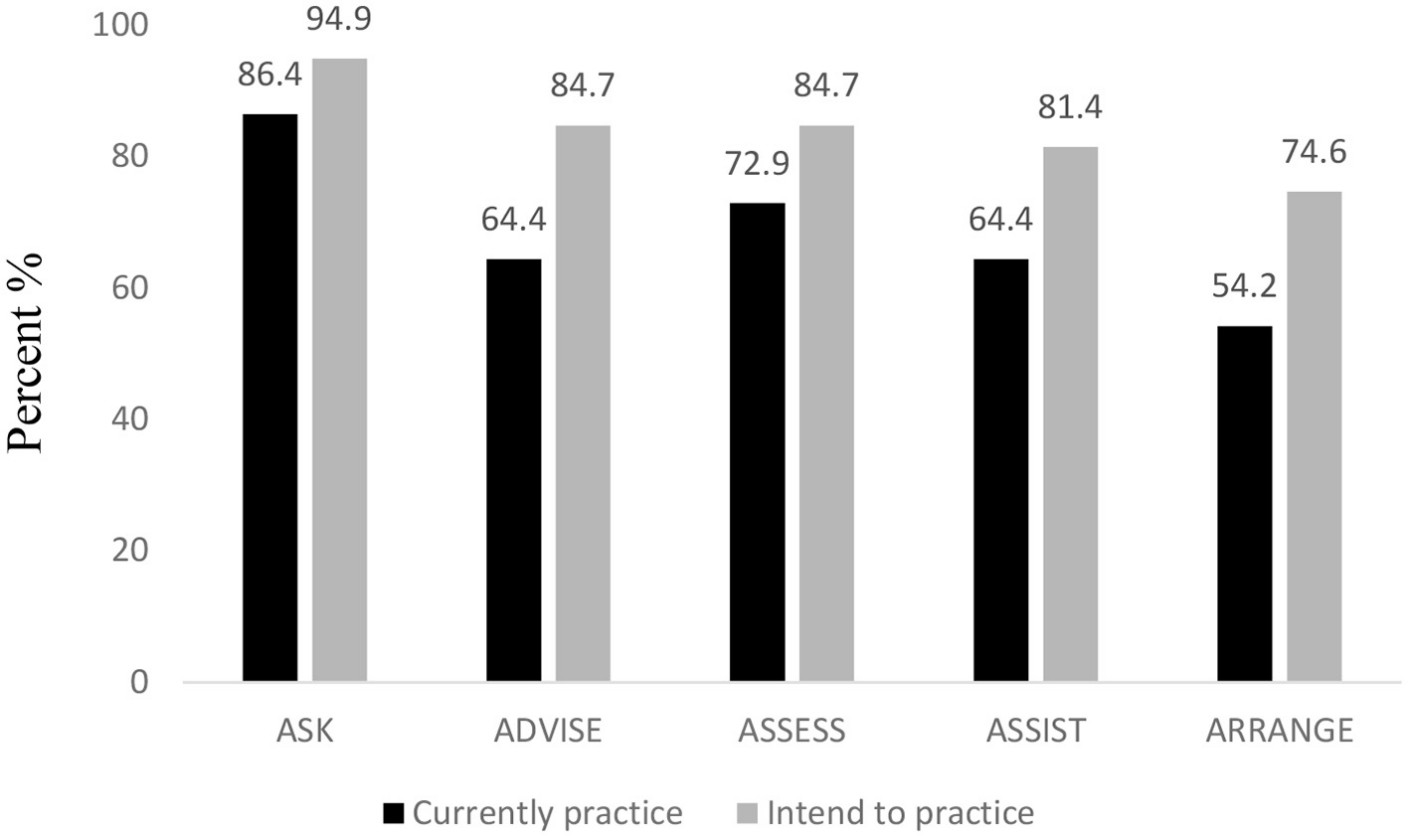
Providers' current and intended practice (occasional/very often) of evidence-based tobacco treatment before and after engaging in the simulated scenarios.

Among participants who responded to both the pre- and post-survey questions (*n* = 59), the proportion of reported current practice (occasional/very often) of evidence-based tobacco treatment was highest for ASK (86.4%), followed by ASSESS (72.9%), followed by ADVISE and ASSIST (64.4% each), and ARRANGE (54.2%). After engaging in the simulated scenario, the proportion of those who intended to practice evidence-based tobacco treatment increased in each component (see Figure 1).

## Discussion

The purpose of this study was to evaluate a pilot intervention designed to improve MHPs' knowledge about tobacco use and mental illnesses and evidence-based practice in tobacco treatment. To our knowledge, ours is the first study to assess the use of animated simulated scenarios to enhance knowledge and intentions to provide tobacco treatment. This evaluation of the intervention yielded acceptable scores, suggesting that the intervention is desirable, acceptable, and applicable to MHPs. Moreover, engagement in the intervention resulted in overall increased knowledge scores, proficiency, and intentions to provide evidence-based practice. These findings provide preliminary support for use of simulated tailored tobacco treatment scenarios as an avenue for provider education and training.

Participants provided moderate to high mean scores on the desirability, acceptability, and applicability scale. Our findings are consistent with a prior qualitative study that used similar scales to evaluate the components of tailored tobacco treatment programs for patients with SSD (23). Furthermore, the lowest quality rating for the use of animation was observed with the SSD scenario and the highest with the ADHD scenario. This difference may be explained in that the SSD scenario was rated primarily by adult providers, whereas the ADHD scenario was rated by mostly pediatric providers. This finding may warrant further qualitative explorations of differences in appeal of scenario delivery format (i.e., cartoon-based animation versus realistic depictions) based on the age of the populations served by the MHPs.

In addition, we found that the simulated scenario intervention resulted in immediate post-test changes in knowledge scores related to tobacco use and treatment in specific MI populations. A previous study found that tobacco treatment training increases knowledge, competency, and self-efficacy in tobacco treatment delivery by MHPs (30). Moreover, a more recent study assessing the use of virtual simulation to enhance counseling skills for alcohol dependence treatment among social work master's level students found that engagement in the simulation-based training resulted in improved self-efficacy and general clinical skills (31). In a similar fashion, our study findings provide some level of validation for the use of animated scenarios to enhance knowledge acquisition and intentions to change practice. Future studies with larger samples are needed to assess the use of these scenarios on a wider scale.

A few important limitations are necessary to properly consider the implications of our findings. First, this pilot study used a pre-post study design with only one post-test after the intervention. This design limits our ability to determine sustained changes in the knowledge acquisition or behavioral intentions observed in our study. Future studies using longitudinal assessments beyond the single post-test may better determine the prolonged impact of the intervention. Second, there was limited representation by counselors and other types of MHPs in the study sample. We had fewer than five providers from a particular specialty evaluating some scenarios. Our goal was to have at least five providers from among prescribers, counselors/ therapists, nurses, and social workers. Due to our recruitment process, the survey link for the evaluation may have been shared by participants to other non-MHPs who were not our main target (e.g., women's health providers evaluating the depression simulated scenario). Future studies should target specific provider groups with adequate samples to better evaluate the intervention effect. Also, the study sample underrepresented individuals from outpatient behavioral health and residential recovery settings. Targeting such sites can improve our knowledge of the impact of these scenarios across different settings. Third, it is important to note that few of the providers had prior training in tobacco treatment and about a fifth were tobacco users. Hence, given that providers who use tobacco are less likely to treat tobacco users (32, 33), our findings may have been affected by the tobacco use behaviors of the participants. Finally, the MHPs in our study were primarily female and were from a single geographic location. Hence, we cannot generalize the findings to other settings. Future studies may incorporate a random sampling of MHPs from different geographic areas to further determine the effectiveness of the scenarios in enhancing knowledge and practices.

In conclusion, ours was the first to evaluate the use of animated scenarios for tobacco treatment among MHPs. Given the exorbitant toll of tobacco use disorders among PMI, it is critical to determine easily accessible and innovative methods to enhance MHPs' training in tobacco treatment delivery. Using animated simulated scenarios of evidence-based treatment may be an option of quick delivery with easy access. Future studies are needed to further evaluate the use of such simulated scenarios across different MHPs and in broader settings. Such studies can yield valuable knowledge to enhance interventions to address the disproportionate tobacco use and disease burden among PMI.

## Data Availability Statement

The raw data supporting the conclusions of this article will be made available by the authors, without undue reservation.

## Ethics Statement

The studies involving human participants were reviewed and approved by University of Kentucky Institutional Review Board. Written informed consent for participation was not required for this study in accordance with the national legislation and the institutional requirements.

## Author Contributions

CO conceived of the study, completed all data analysis, and developed the results section. JO developed the surveys, assisted with the development of the study, reviewed, and revised drafts of the manuscript. SS and BA drafted the introduction, measures section, assisted with drafting, and reviewing the discussion section. LW provided intellectual contribution throughout the manuscript, reviewed, and revised the final drafts. All authors contributed to the article and approved the submitted version.

## Funding

Research reported in this presentation was supported, in part, by the Cabinet for Health and Family Services, Department for Public Health Tobacco Prevention and Cessation Program under Agreement titled Enhancing Tobacco Dependence Treatment in Community Mental Health Centers.

## Author Disclaimer

The content is solely the responsibility of the authors and does not necessarily represent the official views of the Cabinet for Health and Family Services, Department for Public Health, Tobacco Prevention and Cessation Program.

## Conflict of Interest

The authors declare that the research was conducted in the absence of any commercial or financial relationships that could be construed as a potential conflict of interest.

## Publisher's Note

All claims expressed in this article are solely those of the authors and do not necessarily represent those of their affiliated organizations, or those of the publisher, the editors and the reviewers. Any product that may be evaluated in this article, or claim that may be made by its manufacturer, is not guaranteed or endorsed by the publisher.
